# Categorization of species as native or nonnative using DNA sequence signatures without a complete reference library

**DOI:** 10.1002/eap.1914

**Published:** 2019-06-12

**Authors:** Jeremy C. Andersen, Peter Oboyski, Neil Davies, Sylvain Charlat, Curtis Ewing, Christopher Meyer, Henrik Krehenwinkel, Jun Ying Lim, Suzuki Noriyuki, Thibault Ramage, Rosemary G. Gillespie, George K. Roderick

**Affiliations:** ^1^ Department of Environmental Science Policy and Management University of California Berkeley 130 Mulford Hall Berkeley California 94720‐3114 USA; ^2^ Essig Museum of Entomology University of California Berkeley Berkeley California 94720 USA; ^3^ Gump South Pacific Research Station University of California Berkeley Maharepa Moorea French Polynesia; ^4^ Biométrie et Biologie Évolutive UMR CNRS 69622 Villeurbanne France; ^5^ Komohana Research and Extension Center University of Hawai'i at Mānoa Hilo Hawaii 96720 USA; ^6^ Smithsonian Institution Washington D.C. 20013 USA; ^7^ Department of Biogeography Universität Trier Trier Germany; ^8^ Department of Integrated Biology University of California Berkeley 3040 Valley Life Sciences Building Berkeley California 94720 USA; ^9^ Faculty of Agriculture and Marine Science Kochi University Kochi Japan; ^10^ 9 Quartier de la Glacière 29900 Concarneau France

**Keywords:** alien invasive species, biomonitoring, biosecurity, community barcoding, DNA barcoding, metabarcoding, Moorea BioCode

## Abstract

New genetic diagnostic approaches have greatly aided efforts to document global biodiversity and improve biosecurity. This is especially true for organismal groups in which species diversity has been underestimated historically due to difficulties associated with sampling, the lack of clear morphological characteristics, and/or limited availability of taxonomic expertise. Among these methods, DNA sequence barcoding (also known as “DNA barcoding”) and by extension, meta‐barcoding for biological communities, has emerged as one of the most frequently utilized methods for DNA‐based species identifications. Unfortunately, the use of DNA barcoding is limited by the availability of complete reference libraries (i.e., a collection of DNA sequences from morphologically identified species), and by the fact that the vast majority of species do not have sequences present in reference databases. Such conditions are critical especially in tropical locations that are simultaneously biodiversity rich and suffer from a lack of exploration and DNA characterization by trained taxonomic specialists. To facilitate efforts to document biodiversity in regions lacking complete reference libraries, we developed a novel statistical approach that categorizes unidentified species as being either likely native or likely nonnative based solely on measures of nucleotide diversity. We demonstrate the utility of this approach by categorizing a large sample of specimens of terrestrial insects and spiders (collected as part of the Moorea BioCode project) using a generalized linear mixed model (GLMM). Using a training data set of known endemic (*n = *45) and known introduced species (*n *=* *102), we then estimated the likely native/nonnative status for 4,663 specimens representing an estimated 1,288 species (412 identified species), including both those specimens that were either unidentified or whose endemic/introduced status was uncertain. Using this approach, we were able to increase the number of categorized specimens by a factor of 4.4 (from 794 to 3,497), and the number of categorized species by a factor of 4.8 from (147 to 707) at a rate much greater than chance (77.6% accuracy). The study identifies phylogenetic signatures of both native and nonnative species and suggests several practical applications for this approach including monitoring biodiversity and facilitating biosecurity.

## Introduction

The genomics revolution is transforming the studies of conservation biology, ecology, and evolution (Hudson 2008, Allendorf et al. 2010). New affordable sequencing technologies, coupled with decreases in traditional sequencing costs and in the time required to process specimens (see, for example, Pomerantz et al. 2018) have made it possible to sequence DNA from entire communities of individuals (Borisenko et al. 2008, Kress et al. 2009, Cristescu 2014) and allow for the production and publication of genomic information from non‐model organisms from across the tree of life (Ekblom and Galindo 2011). One approach for collecting and analyzing sequence data that has become widely utilized is known as DNA sequence barcoding (also known as “DNA barcoding”; Hebert et al. 2003, Savolainen et al. 2005, Ratnasingham and Hebert 2007). This technique has been used in many contexts; including biodiversity inventories (Janzen et al. 2005), cryptic species discovery (Hebert et al. 2004a), species identification (Hebert et al. 2003, 2004b, Kress et al. 2005), species delimitation (Pons et al. 2006), biomonitoring (Pilgrim et al. 2011), biosecurity (Saunders 2009, Collins et al. 2012, Dejean et al. 2012, Porco et al. 2013, Ashfaq and Hebert 2016, Thomas et al. 2016), for phylogenetic and population genetic studies (Hajibabaei et al. 2007), and for observations of within‐species genetic diversity (e.g., Johnson et al. 2002, Havill et al. 2018). While there are numerous well documented limitations to the uses of DNA barcoding, including when the technique is used to reconstruct ancient evolutionary relationships, when non‐specific amplification is not accounted for, and when fixed intra‐ and inter‐interspecific thresholds are utilized (Moritz and Cicero 2004, Thalmann et al. 2004, DeSalle et al. 2005, Meyer and Paulay 2005, Rubinoff et al. 2006, Buhay 2009, Moulton et al. 2010), one of the core uses for DNA barcoding is the comparison of query sequences to reference DNA sequences to determine the percentage of sequence similarity.

As such, the primary objective for most DNA barcoding projects is to obtain species‐level identifications from specimens that for one reason or another lack a morphologically derived species‐level identification. In order to do so, however, a researcher first requires access to a well‐curated reference library that includes DNA sequences from previously identified species, ideally including all potential species matches. While numerous efforts to produce these reference libraries are currently underway (e.g., Blagoev et al. 2015, 2016, Cardoni et al. 2015, Gwiazdowski et al. 2015, Oliveira et al. 2015, 2016, Park et al. 2015, Savage et al. 2015, Warne et al. 2015, Xu et al. 2015, Iftikhar et al. 2016, Lobo et al. 2016, Rimet et al. 2016, Yang et al. 2016, Bell et al. 2017, Pennisi 2017), until complete reference libraries exist for all taxonomic groups and for all geographic locations, the comparison of a query sequence to sequences in public reference libraries such as the Barcode of Life Database (BOLD; Ratnasingham and Hebert 2007) or GenBank (Benson et al. 2013), or a researcher's private database, will result in one of the following four outcomes: (1) a high‐percentage match to a specimen with a species‐level identification, (2) a low‐percentage match to a specimen with a species‐level identification, (3) a high‐percentage match to a sequence from an unidentified specimen (usually noted as some variation of “Undet sp”), or (4) a low‐percentage match to a sequence from an unidentified specimen. In the case of the first outcome, the investigator can use the species‐level identification to inform their research/management decisions, though one needs to be cautious when utilizing these identifications in public reference libraries due to the many known misidentifications (Buhay 2009, Tixier et al. 2011). In the case of the last three outcomes, after the expenditure of time, money, and resources, the specimen in question remains unidentified, and for most research objectives provides little utility, though in some instances higher level categorizations (i.e., genus, family, order) may be obtained and be sufficient. While the collection of DNA barcode sequences from unidentified specimens provides useful genomic data, unfortunately at the same time that DNA barcoding techniques are being used with increasing frequency to guide management decisions (e.g., Park et al. 2015), particularly for the identification of alien invasive species (Saunders 2009, Dejean et al. 2012, Porco et al. 2013, Ashfaq and Hebert 2016, Thomas et al. 2016), the taxonomists needed to provide the foundational species‐level identifications to create reference libraries are themselves becoming endangered species (Gotelli 2004, Agnarsson et al. 2007, Wägele 2011, Wheeler 2014)!

The Moorea BioCode project was established, in part, to address the coupled needs for increased sampling of underrepresented taxa in biodiverse tropical regions and hands‐on training for taxonomic specialists (Field and Davies 2015). As such it represents the first comprehensive inventory of all non‐microbial life in a complex tropical ecosystem (Meyer 2017). The Moorea BioCode project has resulted in the DNA sequencing of both terrestrial and aquatic organisms from a variety of diverse taxonomic groups (Nitta 2008, Nitta et al. 2011, Diaz et al. 2013, Bonnet and Lotufo 2015, Leray et al. 2015, Ramage et al. 2017). However, as for many other community sequencing projects, the effort collected specimens (>20,000), and obtained DNA sequences (~5,000) from our focal taxonomic groups (spiders and insects) at a rate much faster than the specimens could be assigned to morphologically based species‐level identifications or even species‐level identifications through traditional DNA barcoding approaches.

This challenge in identification prompted the question as to whether one could apply basic concepts from invasion biology to categorize unidentified specimens and/or species whose biogeographic status (i.e., native or nonnative) is unclear. For example, if one assumes that the establishment of most introduced species into novel habitats results in genetic bottlenecks (Dlugosch and Parker 2008), then we might predict that DNA sequences from nonnative species would have lower levels of intraspecific diversity compared to those from native species. Similarly, if nonnative species establish because they can utilize a novel niche (MacDougall et al. 2009), avoid detection from native natural enemies (Keane and Crawley 2002), are uniquely superb dispersers (Gillespie et al. 2012), and/or are more genetically distinct from native members of the community (Strauss et al. 2006), it is likely that the genetic distance to the nearest neighbor species in the collection might be greater for nonnative species then for native species (i.e., phylogenetic distance; see review by Gallien and Carboni 2017). Because the native/nonnative status of a species is often difficult to determine in natural communities, here we propose to use a training data set of known introduced and known endemic species with the assumption that introduced and endemic species might be at opposite ends of the native/nonnative spectrum and that if the above measures of genetic diversity are distinct between native and nonnative species, that they should be even more so between endemic and introduced species. Thus, the objectives of the current study were (1) to determine whether metrics of within species similarities and between species distances differ between known endemic and known introduced species in French Polynesia, (2) to construct a statistical model based on measures of genetic differences between, and genetic variation within known endemic and known introduced species, (3) to use the model to predict the likely native or likely nonnative status of unidentified and uncategorized specimens, and (4) to explore possible ecological applications for the method.

## Methods

### Field work, specimen sampling, and laboratory protocols

Specimens were collected in the field, assigned a preliminary identification to the lowest possible taxonomic level, and entered in the BioCode Field Information Management System (FIMS; Deck et al. 2012). When possible, taxonomic specialists then provided more refined genus‐ or species‐level identifications. Community sampling, DNA extraction, and barcode sequencing methods of specimens for a variety of taxonomic groups as part of the Moorea BioCode project are described elsewhere (Nitta 2008, Nitta et al. 2011, Diaz et al. 2013, Bonnet and Lotufo 2015), including methods specific for insects (Ramage et al. 2017). For each specimen, a DNA barcode sequence (corresponding to the 5′ region of cytochrome oI) was generated following standard protocols and recorded by the BioCode Laboratory Information Management System (LIMS; Parker et al. 2012), a free plugin developed for the Geneious software system (Kearse et al. 2012; *available online*).^1^ All BioCode sequences and their associated metadata are available via The Genomic Observatories Metadatabase (Deck et al. 2017; data *available online*).^2^

### Data filtering

As a first filter, sequences of less than 300 base pairs (b.p.) were removed from the data set to ensure that included sequences had sufficient overlap to allow for pairwise distance estimates in downstream analyses. Each sequence was compared to published sequences in the NCBI GenBank database using the blastn search algorithm (Altschul et al. 1990) run locally using the command‐line interface and compared to the nt sequence database (downloaded 11 July 2016) with all default summary statistics retained in tab‐delimited format for the top‐match sequence, except that we also retained the taxids number for the top‐math accession. The taxids number was then used to query the NCBI Taxonomy Database to provide higher level taxonomic information using a custom interactive python script blast2taxoninfo.py (H. Krehenwinkel, G. de Kerdrel, J.C. Andersen, and R. Gillespie, unpublished data). The order‐level morphological identification of each specimen was then compared to the order‐level assignment from the top‐match sequence in the NCBI GenBank Database, and any specimens for which there was disagreement between morphological‐based order assignments and the order assignment for the top‐match sequence were considered as “contaminant” sequences and removed from analyses. DNA sequences, locality collection information, field collection identification, and NCBI top‐match information for each retained specimen are provided in Data S1: CollectionInformation.csv.

### Species delineation and categorization

For the spiders and for each order of insect, unique alignments were generated using the nucleotide alignment software MUSCLE (Edgar 2004) as implemented through the EMBL‐EBI web service (Li et al. 2015). Alignments were subsequently refined in Geneious using the Translational Align sub‐option for the Multiple Alignment tool based on visual examinations of the reading frame prior to refinement using the Invertebrate Mitochondrial genetic code. Refined alignments were inspected and edited by hand when necessary, and further filtered to remove any sequences that did not have >100 b.p. of sequence overlap with every other sequence in the alignment. Specimens were then assigned to species‐groups using Automatic Barcode Gap Discovery (ABGD; Puillandre et al. 2012) using the default values with all partitions and tree files were output using the ‐a setting. While these groups are technically operational taxonomic units (OTUs), for simplification, we will refer to these groups as “species,” though we acknowledge that “species” may not be appropriate in all instances. To determine the optimal delineation scheme from ABGD, we then examined the slopes for the results of the number of species identified using the Recursive Partitions and the Initial Partitions and the prior intraspecific divergence, and for each order chose the value (*P*) that represented the intercept of these two slopes.

Using the species‐group assignments identified above, we then updated specimen identifications using a two‐step approach. First, for any species from which one or more specimens had a collections‐based species‐level identification, that identification was applied to all other unidentified specimens assigned to the same species. Second, the labels for the remaining species that lacked species‐level identifications were updated using the NCBI GenBank best‐match subject sequences as follows: for any species for which a specimen had a ≥97% match to a published sequence with a species‐level identification, that species was assigned the species‐level identification from the published sequence; for any species for which a specimen had a ≥90 but <97% match to a published sequence with a species‐ or genus‐level identification, that species was assigned the genus‐level identification from the published sequence; for any species for which a specimen had a ≥80% but <90% match to published sequence with a species‐, genus‐, or family‐level identification, that species was assigned the family‐level identification sequence from the published sequence; the taxonomic IDs for all remaining species were then updated to include the order name followed by “sp.” (e.g., Diptera sp.). The original specimen identifications, the species‐group to which they were assigned by ABGD, the updated specimen identifications, and whether those updates were based on morphological or molecular methods are provided in Data S1: CollectionInformation.csv.

### Biogeographical status categorization

For each species for which we could obtain a species‐level identification, we then assigned that species the biogeographical categorizations presented for it by Ramage (2017). The categorizations are provided in Data S1: RamageChecklist.csv.

### Model development

For each order, a distance matrix based on that order's nucleotide alignment was generated. Using a custom script (provided in Data S1: CustomRscript.R) written in the statistical language R v 3.1.3 (R Core Team 2014), we then used the distance matrix to estimate the average distance between individuals within a species (i.e., average similarity) and average distance between individuals of nearest‐neighbor species (i.e., average distance). The data was then subset to include a training data set that included the above statistics only for species with species‐level identifications and a biogeographical categorization of either endemic or introduced in Ramage (2017), and the full data set that included the statistics for all species regardless of whether or not they had a species‐level identification or a known status of endemic/introduced. Preliminary analyses based on Welch's two‐sample *t* test using the training data set (Appendix S1: Fig. S1) indicated that there were highly significant differences between endemic and introduced species based on measures of average distance (*t* = 5.897, df = 114.1, *P* value < 0.0001), while there were no significant differences based on measures average similarity (*t* = 0.958, df = 112.5, *P* = 0.34). We then constructed four generalized linear mixed models (GLMMs), using the R package lme4 (Bates et al. 2015) with species categorization as the response variable coded as binomials (i.e., endemic = 1, and introduced = 0), average similarity and/or average distance as fixed effects, and we included order as a random effect because we were concerned that the model might have better fit for some orders than others. Models were then compared based on AIC_c_ scores using the R package AICcmodavg (Mazerolle 2017) to determine the model with the best fit for the data set. Using the loadings from the best‐fit GLMM calculated using the training data set, we then assigned scores from 0 to 1 to each species in the full data set using the predict command in R. Ninety‐five percent confidence intervals (CI) from the GLMM assignment scores for endemic and introduced species in the training data set were then calculated (mean ± 1.96 × SE). Any species in the full data set with a predicted value ≤ the upper 95% CI for introduced species was assigned the label “GLMM–Nonnative,” any species in the full data set with a predicted value ≥ the lower 95% CI for endemic species was assigned the label “GLMM–Native,” and any species in the full data set with a predicted value that fell between the two CIs was assigned the label “GLMM–Undet.” Error rates were then calculated globally and for each order by recording the percentage of species in the training data set that had a known endemic/introduced categorization but were incorrectly assigned the opposite native or nonnative status based on the GLMM.

## Results

### Specimen sampling and data filtering

After removal of sequences of <300 base pairs, and the removal of sequences identified as “contaminants” based on order‐level mismatches between the morphological identifications and the order‐level identification for their best‐match sequence in GenBank, the final data set included cytochrome oxidase I (COI) sequences from 4,663 specimens. Species‐level identifications were given to 2,172 specimens using a combination of morphologically based identifications, identifications published in Ramage et al. (2017), or identifications based on matches of ≥97% similarity to a published sequence in the NCBI GenBank database.

### Species delimitation and categorization

Species delimitation based on ABGD estimated the presence of 1,288 species (Araneae *n *=* *52, Blattodea *n *=* *8, Coleoptera *n *=* *141, Diptera *n = *312, Hemiptera *n = *204, Hymenoptera *n *=* *191, Lepidoptera *n *=* *328, Neuroptera *n = *5, Odonata *n *=* *7, Psocoptera *n *=* *34, Thysanoptera *n *=* *6). Of these, 280 species are present in the checklist for French Polynesia published by Ramage (2017), including 42 endemic species and 98 introduced species.

### Model development

AIC_c_ scores for the four GLMM models based on between species distances are presented in Table 1. The model with the lowest AIC_c_ score was determined to be

**Table 1:** Model choice

Model	LL	*K*	AIC_c_	∆AIC_c_	*w*
Average similarity + average distance + (1|order)	−63.90	4	136.15		0.71
Average similarity × average distance + (1|order)	−63.89	5	138.30	2.16	0.24
Average distance + (1|order)	−67.58	3	141.38	5.23	0.05
Average similarity + (1|order)	−74.80	3	155.77	19.62	0

*Note*

Akaike information criterion corrected for sample size (AIC_c_) scores for GLMM models including log‐likelihood (LL) values, number of parameters (*K*), AIC_c_ score, change in AIC_c_ scores from one model to the next (∆AIC_c_), and the models weight (*w*).

$$\begin{array}{cc} \text{Classification}\;∼\; & \text{Average Similarity}\\ & +\text{Average Distance}+(1|\text{Order}) \end{array}$$

For introduced species in the training data set, the mean GLMM value was 0.2582 with a 95% confidence interval of 0.2291–0.2873. For endemic species in the training data set, the mean value was 0.3839 with a 95% confidence interval of 0.3444–0.4233. Mean values and 95% confidence intervals for individual orders are presented in Table 2.

**Table 2:** GLMM scores for known introduced and endemic species

Order	Introduced	Endemic
Araneae	0.5695 (0.5601,0.5788)	0.5624 (0.5477,0.5772)
Blattodea		
Coleoptera	0.1341 (0.1001,0.1681)	0.1824 (0.1639,0.2009)
Diptera	0.3267 (0.2887,0.3647)	0.3518 (0.3138,0.3899)
Hemiptera	0.1941 (0.1360,0.2522)	0.2210 (0.0791,0.3629)
Hymenoptera	0.1959 (0.1601,0.2317)	0.2305 (0.0257,0.4352)
Lepidoptera	0.3546 (0.3148,0.3945)	0.3845 (0.3439,0.4250)
Odonata		0.3841 (0.3350,0.4331)
Psocoptera		
Thysanoptera	0.0511 (0.0465,0.0557)	
Grand total	0.2582 (0.2291,0.2873)	0.3839 (0.3444,0.4233)

*Note*

Values are reported as the mean value for species in each order followed by the lower 99% CI and the upper 99% CI in parentheses.

### GLMM‐based biogeographical categorizations and error‐rates

**Figure 1:**
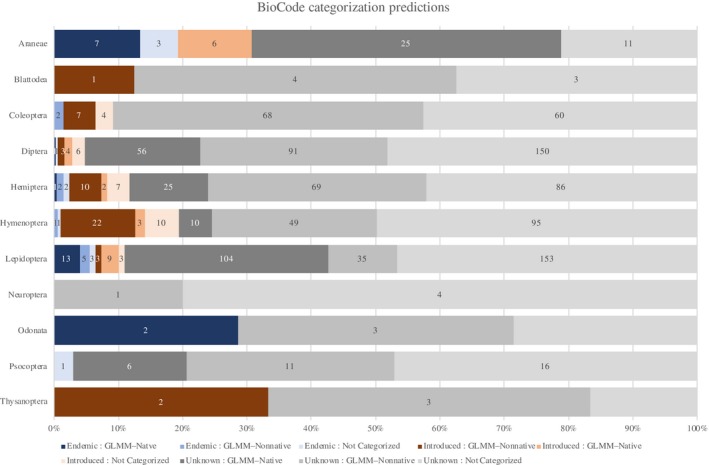
Comparison of the biogeographical categorizations based on Ramage (2017) or based on GLMM predictions. Categorizations are presented so that the biogeographic assignment in Ramage (2017) is before the colon and the biogeographic assignment based on the GLMM predictions is presented after the colon. Categorizations follow Table 3, except that two categories for the GLMM predictions (Undet and NA) have been combined into a sing category here labeled “Not Categorized.”

Using the GLMM value thresholds described above, specimens in the full data set were assigned labels of either GLMM–Nonnative (*n *=* *2,040), GLMM–Native (*n *=* *1,457), or GLMM–Undet (*n *=* *639). For an additional 509 specimens, we were unable to calculate a GLMM value as they were the only representatives of their respective species. Two‐hundred and seventy‐three species were predicted to be likely native, 393 species were predicted to be likely nonnative, 100 species were unassigned as their GLMM values fell between the 95% confidence intervals for known introduced and endemic species. Among endemic species, 24 species were predicted to be likely native, 10 species were predicted to be likely nonnative, five species fell between the 95% CIs for endemic and introduced species, and six species were assigned “NA,” as they were singleton species (total unassigned, 22.2%). Among introduced species, 49 species were predicted to be likely nonnative, 23 species were assigned as likely native, eight species fell between the 95% CIs for endemic and introduced species, and 22 species were assigned “NA” as they were singleton species (total unassigned 22.6%). GLMM scores for all species are presented in Data S1: SummaryGeneticInformation.csv and summarized for each order including error rates in Table 3 and presented graphically in Fig. 1.

**Table 3:** Numbers of endemic, introduced, and unknown species in a data set based on species‐level identifications and the categorizations in Ramage (2017)

Training	Introduced					Endemic					Unknown			
Predicted	Native	Nonnative	Undet	NA	Error (%)	Native	Nonnative	Undet	NA	Error (%)	Native	Nonnative	Undet	NA
Araneae	6				100	7			3	0	25			11
Blattodea		1			0							4		3
Coleoptera		7		4	0		2			100		68	3	57
Diptera	3	4	3	3	23.08	1		1		0	56	91	29	121
Hemiptera	2	10	2	5	10.53	1	2		2	40	25	69	7	79
Hymenoptera	3	22	1	9	8.57		1	1		50	10	49	12	83
Lepidoptera	9	3	2	1	60	13	5	3		23.81	104	35	44	109
Neuroptera												1	3	1
Odonata						2				0		3		2
Psocoptera									1	0	6	11	2	14
Thysanoptera		2			0							3		1
Grand total	23	49	8	22	22.55	24	10	5	6	22.22	226	334	100	481

*Notes*

Below each categorization, the numbers of species predicted to be likely endemic, likely introduced, as well as the number of species that could not be categorized by the model either because the GLMM value fell between the 95% Confidence Interval for Introduced and Endemic species (Undet) or because the species was represented by only a single specimen (NA), are shown along with the error rate (i.e., the percentage of species assigned as likely introduced for endemic species, or likely endemic for introduced species). The global error rate for each order is provided in the rightmost column.

### Within and between species variation

Based on GLMM classifications, the method provides estimates of a phylogenetic signature of both native and nonnative species (Data S1: SummaryGeneticInformation.csv). The mean values predicted by the GLMM for within species variation (i.e., average similarity) for native species was 0.0076 ± 0.0006 and for nonnative species was 0.0095 ± 0.0010, and for between species variation (i.e., average distance) for native species was 0.0531 ± 0.0015 and for nonnative species was 0.1788 ± 0.0025. GLMM estimates are presented in Data S1: SummaryGeneticInformation.csv.

## Discussion

DNA sequences are increasingly being used for the rapid quantification of biodiversity (Smith and Fisher 2009), and for some taxonomic groups may provide a more accurate overview of species diversity than traditional (i.e., morphological) methods (Teasdale et al. 2013, Stein et al. 2014). While these approaches can be used to highlight biogeographic regions that are at risk due to anthropogenic disturbances (Bilgin et al. 2016) and have great potential for use by ecologists, evolutionary, and conservation biologists (Valentini et al. 2009, Kress et al. 2015), frequently the utility of these methods are constrained by the presence of incomplete reference libraries (Elias et al. 2007, Gonzalez et al. 2009). Here we have presented a technique that can be utilized by any community DNA sequencing project to rapidly categorize unidentified specimens or specimens from species whose biogeographic status is unknown as representing either likely native or nonnative species. For example, using a traditional reference library approach (i.e., combining morphological identifications with matches to sequences in public databases with species‐level identifications), we were able to categorize 794 of the 4,663 sequenced specimens (*n *=* *147 species) as being either endemic or introduced species. In contrast, using our GLMM approach, 3,497 of the 4,663 sequenced specimens (*n *=* *707 species) could be categorized as either likely native or likely nonnative. We discuss several possible applications for this approach below.

### Applicability for biosecurity and quarantine monitoring

Biosecurity, particularly in regards to the identification of specimens in quarantine situations, has benefited greatly from the use of DNA sequencing approaches to identify known pests (Armstrong and Ball 2005, Ball and Armstrong 2006) and exotic species (Pejovic et al. 2016, Ardura and Planes 2017), and in some locations national, and even international, programs are being established to integrate DNA sequencing and biosecurity efforts (e.g., Hodgetts et al. 2016). Yet, DNA approaches fail to identify potential quarantine species when previously sequenced individuals of that species are missing from the reference library. While previous research has shown that the utility of DNA sequences to improve biosecurity monitoring can further be increased through the integration of multiple forms of analysis (Collins et al. 2012), other approaches that might improve the identification of these “known‐unknown” and “unknown‐unknown” specimens that are absent from reference libraries includes the use of multi‐species multi‐locus coalescent (Dowton et al. 2014, though see Collins and Cruickshank 2014 for a critique) or methods that utilize molecular clocks coupled with population genetics to determine whether a species arrived in a location prior to human colonization (Pisa et al. 2015). While our approach cannot provide species identifications for specimens absent from the reference library, it can be used to prioritize which specimens are examined by trained taxonomists. Under our approach, the time required to provide a morphological identification could be streamlined by reducing the number of specimens and prioritizing those specimens (based on their GLMM scores, for example) that a trained taxonomist examines in a given day. In addition, by working in concert with trained specialists, when species‐level assignments are provided by the taxonomist, the sequence data for the specimen and its associated metadata could then be used to update the reference library and the GLMM model to improve future predictions. However, further validation of the approach and explorations of what are acceptable error rates is required, preferably from multiple localities and from multiple taxonomic groups.

### Limitations and future directions

While we are hopeful that our approach can be broadly applied to both biodiversity and biosecurity issues, we need to highlight several limitations that must be addressed before it can be used more widely. The greatest limitation to our approach is the requirement for community‐wide sampling, preferably obtained using standardized collection methods from random localities: methods that might miss rare or cryptic species and result in the collection of large numbers of “common” specimens. A second challenge for tropical ecosystems comes from the lack of endemic species in existing reference libraries. Because it is more likely that cosmopolitan species will have DNA sequences in public reference libraries, our model (and others generated for different study regions) might be inherently biased towards the categorization of introduced species. In certain circumstances, however, species‐level identifications might not be necessary for endemic lineages where all members of a genus (or higher taxonomic categorization) are endemic to the focal locality, and thus the generic (or higher) assignments could be utilized to assign those specimens as endemic when building the GLMM. A third limitation comes in regards to picking a biologically meaningful cutoff for the GLMM scores to differentiate endemic and introduced species. Here, we adopted a conservative approach, and only assigned species as likely native or likely nonnative based on 95% CIs from known endemic and known introduced species. This resulted in a large number of specimens (*n *=* *639) and species (*n *=* *113) being uncategorized as their GLMM scores fell between the upper 95% CI for introduced species and the lower 95% CI for endemic species. One possible solution for determining biologically meaningful cutoffs is the use of machine learning, an approach we are currently exploring. A fourth potential limitation of our model is in regards to the effects of multiple introductions. Certain taxonomic groups are likely to be introduced repeatedly, and often from multiple locations, thus resulting in additive genetic variation and or presenting short branching patterns similar to endemic species that might influence our predictive model. A fifth potential limitation of our model is that it requires multiple samples from each species. In our sample, 509 specimens represented the only examples of their respective species, and thus we were unable to calculate within species diversity. A sixth potential limitation of our model is that it may work better for certain orders than for others. For example, while unclassified species from most orders were assigned to a mix of both native and nonnative categories, for several orders all species were classified as either native or nonnative. Whether this is the result of uneven sampling, too few species in the training data set, and/or different rates of evolution, is unknown, but we expect that in the future order‐specific analyses will likely improve the overall predictive power of the model. Finally, and perhaps the greatest limitation of the approach, it is unclear to which ecosystems our approach can most appropriately be applied. Evolutionary biologists have long focused on island ecosystems due to their isolation, their high rates of endemism, and their suitability for observing the effects of evolution (Darwin 1859, Wallace 1880, Gillespie and Roderick 2014, Cressey 2015). However, whether our approach is transferable to continental systems, where species mixing occurs at higher rates and phylogenetic patterns may be more diffuse, is unclear, though we enthusiastically encourage the testing of our method for categorizing community barcode sequences collected in additional settings.

## Data Availability

Data are available from the Genomic Observatories Metadatabase: https://geome-db.org/workbench/overview?projectId=75

## Notes

1
http://software.mooreabiocode.org/

2
https://geome-db.org/workbench/overview?projectId=75

## References

[eap1914-bib-0001] Agnarsson, I. , M. Kuntner , and A. Paterson . 2007 Taxonomy in a changing world: seeking solutions for a science in crisis. Systematic Biology 56:531–539. 17562477 10.1080/10635150701424546

[eap1914-bib-0002] Allendorf, F. W. , P. A. Hohenlohe , and G. Luikart . 2010 Genomics and the future of conservation genetics. Nature Reviews Genetics 11:697–709. 10.1038/nrg2844 20847747

[eap1914-bib-0003] Altschul, S. F. , W. Gish , W. Miller , E. W. Myers , and D. J. Lipman . 1990 Basic local alignment search tool. Journal of Molecular Biology 215:403–410. 2231712 10.1016/S0022-2836(05)80360-2

[eap1914-bib-0004] Ardura, A. , and S. Planes . 2017 Rapid assessment of non‐indigenous species in the era of the eDNA barcoding: A Mediterranean case study. Estuarine Coastal and Shelf Science 188:81–87.

[eap1914-bib-0005] Armstrong, K. F. , and S. L. Ball . 2005 DNA barcodes for biosecurity: invasive species identification. Philosophical Transactions of the Royal Society B: Biological Sciences 360:1813–1823. 10.1098/rstb.2005.1713 PMC1609225 16214740

[eap1914-bib-0006] Ashfaq, M. , and P. D. N. Hebert . 2016 DNA barcodes for bio‐surveillance: regulated and economically important arthropod plant pests. Genome 59:933–945. 27753511 10.1139/gen-2016-0024

[eap1914-bib-0007] Ball, S. L. , and K. F. Armstrong . 2006 DNA barcodes for insect pest identification: a test case with tussock moths (Lepidoptera: Lymantriidae). Canadian Journal of Forest Research 36:337–350.

[eap1914-bib-0008] Bates, D. , M. Maechler , B. Bolker , and S. Walker . 2015 Fitting linear mixed‐effects models using lme4. Journal of Statistical Software 67:1–48.

[eap1914-bib-0009] Bell, K. L. , V. M. Loeffler , and B. J. Brosi . 2017 An rbcl reference library to aid in the identification of plant species mixtures by DNA metabarcoding. Applications in Plant Sciences 5:1600110. 10.3732/apps.1600110 PMC5357121 28337390

[eap1914-bib-0010] Benson, D. A. , M. Cavanaugh , K. Clark , I. Karsch‐Mizrachi , D. J. Lipman , J. Ostell , and E. W. Sayers . 2013 GenBank. Nucleic Acids Research 41:D36–D42. 23193287 10.1093/nar/gks1195 PMC3531190

[eap1914-bib-0011] Bilgin, R. , N. Ebeoglu , S. Inak , M. A. Kirpik , J. J. Horns , and C. H. Sekercioglu . 2016 DNA barcoding of birds at a migratory hotspot in eastern Turkey highlights continental phylogeographic relationships. PLoS ONE 11:e0154454. 27304877 10.1371/journal.pone.0154454 PMC4909268

[eap1914-bib-0012] Blagoev, G. A. , J. deWaard , and P. D. N. Hebert . 2015 Building a DNA barcode reference library for Canadian spiders (Araneae). Genome 58:197. 10.1111/1755-0998.12444 26175299

[eap1914-bib-0013] Blagoev, G. A. , J. R. deWaard , S. Ratnasingham , S. L. deWaard , L. Q. Lu , J. Robertson , A. C. Telfer , and P. D. N. Hebert . 2016 Untangling taxonomy: a DNA barcode reference library for Canadian spiders. Molecular Ecology Resources 16:325–341. 26175299 10.1111/1755-0998.12444

[eap1914-bib-0014] Bonnet, N. Y. K. , and T. M. C. Lotufo . 2015 Description of *Ascidia paulayi* sp nov (Phlebobranchia: Ascidiidae) from French Polynesia, with a discussion about the *Ascidia sydneiensis* Stimpson, 1855 group. Zootaxa 3994:283–291. 26250274 10.11646/zootaxa.3994.2.8

[eap1914-bib-0015] Borisenko, A. V. , B. K. Lim , N. V. Ivanova , R. H. Hanner , and P. D. N. Hebert . 2008 DNA barcoding in surveys of small mammal communities: a field study in Suriname. Molecular Ecology Resources 8:471–479. 21585824 10.1111/j.1471-8286.2007.01998.x

[eap1914-bib-0016] Buhay, J. E. 2009 “COI‐like” sequences are becoming problematic in molecular systematic and DNA barcoding studies. Journal of Crustacean Biology 29:96–110.

[eap1914-bib-0017] Cardoni, S. , R. Tenchini , I. Ficulle , R. Piredda , M. C. Simeone , and C. Belfiore . 2015 DNA barcode assessment of Mediterranean mayflies (Ephemeroptera), benchmark data for a regional reference library for rapid biomonitoring of freshwaters. Biochemical Systematics and Ecology 62:36–50.

[eap1914-bib-0018] Collins, R. A. , and R. H. Cruickshank . 2014 Known knowns, known unknowns, unknown unknowns and unknown knowns in DNA barcoding: a comment on Dowton *et al*.. Systematic Biology 63:1005–1009. 25116917 10.1093/sysbio/syu060

[eap1914-bib-0019] Collins, R. A. , K. F. Armstrong , R. Meier , Y. G. Yi , S. D. J. Brown , R. H. Cruickshank , S. Keeling , and C. Johnston . 2012 Barcoding and border biosecurity: identifying Cyprinid fishes in the aquarium trade. PLoS ONE 7:e28381. 22276096 10.1371/journal.pone.0028381 PMC3262790

[eap1914-bib-0020] Cressey, D. 2015 Tropical paradise inspires virtual ecology lab. Nature 517:255–256. 25592514 10.1038/517255a

[eap1914-bib-0021] Cristescu, M. E. 2014 From barcoding single individuals to metabarcoding biological communities: towards an integrative approach to the study of global biodiversity. Trends in Ecology & Evolution 29:566–571. 25175416 10.1016/j.tree.2014.08.001

[eap1914-bib-0022] Darwin, C. 1859 On the origin of species by means of natural selection, or preservation of favoured races in the struggle for life. John Murray, London, UK. PMC5184128 30164232

[eap1914-bib-0023] Deck, J. , J. Gross , S. Stones‐Havas , N. Davies , R. Shapley , and C. Meyer . 2012 Field information management systems for DNA barcoding Pages 255–267 *in* Kress W. and Erickson D., editors. DNA barcodes. Methods in molecular biology (methods and protocols). Volume 858. Humana Press, Totowa, New Jersey, USA. 10.1007/978-1-61779-591-6_12 22684960

[eap1914-bib-0024] Deck, J. , M. R. Gaither , R. Ewing , C. E. Bird , N. Davies , C. Meyer , C. Riginos , R. J. Toonen , and E. D. Crandall . 2017 The genomic observatories metadatabase (GeOMe): A new repository for field and sampling event metadata associated with genetic samples. PLoS Biology 15:e2002925. 28771471 10.1371/journal.pbio.2002925 PMC5542426

[eap1914-bib-0025] Dejean, T. , A. Valentini , C. Miquel , P. Taberlet , E. Bellemain , and C. Miaud . 2012 Improved detection of an alien invasive species through environmental DNA barcoding: the example of the American bullfrog *Lithobates catesbeianus* . Journal of Applied Ecology 49:953–959.

[eap1914-bib-0026] DeSalle, R. , M. G. Egan , and M. Siddall . 2005 The unholy trinity: taxonomy, species delimitation and DNA barcoding. Philosophical Transactions of the Royal Society B 360:1905–1916. 10.1098/rstb.2005.1722 PMC1609226 16214748

[eap1914-bib-0027] Diaz, M. C. , R. W. Thacker , N. E. Redmond , K. O. Matterson , and A. G. Collins . 2013 Phylogenetic novelties and geographic anomalies among tropical Verongida. Integrative and Comparative Biology 53:482–494. 23624868 10.1093/icb/ict033

[eap1914-bib-0028] Dlugosch, K. M. , and I. M. Parker . 2008 Founding events in species invasions: genetic variation, adaptive evolution, and the role of multiple introductions. Molecular Ecology 17:431–449. 17908213 10.1111/j.1365-294X.2007.03538.x

[eap1914-bib-0029] Dowton, M. , K. Meiklejohn , S. L. Cameron , and J. Wallman . 2014 A preliminary framework for DNA barcoding, incorporating the multispecies coalescent. Systematic Biology 63:639–644. 24682413 10.1093/sysbio/syu028

[eap1914-bib-0030] Edgar, R. C. 2004 MUSCLE: multiple sequence alignment with high accuracy and high throughput. Nucleic Acids Research 32:1792–1797. 15034147 10.1093/nar/gkh340 PMC390337

[eap1914-bib-0031] Ekblom, R. , and J. Galindo . 2011 Applications of next generation sequencing in molecular ecology of non‐model organisms. Heredity 107:1–15. 21139633 10.1038/hdy.2010.152 PMC3186121

[eap1914-bib-0032] Elias, M. , R. I. Hill , K. R. Willmott , K. K. Dasmahapatra , A. V. Z. Brower , J. Malllet , and C. D. Jiggins . 2007 Limited performance of DNA barcoding in a diverse community of tropical butterflies. Proceedings of the Royal Society B 274:2881–2889. 17785265 10.1098/rspb.2007.1035 PMC3227132

[eap1914-bib-0033] Field, D. , and N. Davies . 2015 BioCode: the new age of genomics. Oxford University Press, Oxford, UK.

[eap1914-bib-0034] Gallien, L. , and M. Carboni . 2017 The community ecology of invasive species: where are we and what's next? Ecography 40:335–352.

[eap1914-bib-0035] Gillespie, R. , and G. Roderick . 2014 Evolution: geology and climate drive diversification. Nature 509:297–298. 24828187 10.1038/509297a

[eap1914-bib-0036] Gillespie, R. G. , B. G. Baldwin , J. M. Waters , C. I. Fraser , R. Nikula , and G. K. Roderick . 2012 Long‐distance dispersal: a framework for hypothesis testing. Trends in Ecology & Evolution 27:47–56. 22014977 10.1016/j.tree.2011.08.009

[eap1914-bib-0037] Gonzalez, M. A. , C. Baraloto , J. Engel , S. A. Mori , P. Petronelli , B. Riera , A. Roger , C. Thebaud , and J. Chave . 2009 Identification of Amazonian trees with DNA barcodes. PLoS ONE 4:e7483. 19834612 10.1371/journal.pone.0007483 PMC2759516

[eap1914-bib-0038] Gotelli, N. J. 2004 A taxonomic wish–list for community ecology. Philosophical Transactions of the Royal Society B 359:585. 10.1098/rstb.2003.1443 PMC1693344 15253346

[eap1914-bib-0039] Gwiazdowski, R. A. , R. G. Foottit , H. E. L. Maw , and P. D. N. Hebert . 2015 The Hemiptera (Insecta) of Canada: constructing a reference library of DNA barcodes. PLoS ONE 10:e0125635. 25923328 10.1371/journal.pone.0125635 PMC4414572

[eap1914-bib-0040] Hajibabaei, M. , G. A. C. Singer , P. D. N. Hebert , and D. A. Hickey . 2007 DNA barcoding: how it complements taxonomy, molecular phylogenetics and population genetics. Trends in Genetics 23:167–172. 17316886 10.1016/j.tig.2007.02.001

[eap1914-bib-0041] Havill, N. P. , S. D. Gaimari , and A. Caccone . 2018 Cryptic east‐west divergence and molecular diagnostics for two species of silver flies (Diptera: Chamaemyiidae: Leucopis) from North America being evaluated for biological control of hemlock woolly adelgid. Biological Control 121:23–29.

[eap1914-bib-0042] Hebert, P. D. N. , A. Cywinska , S. L. Ball , and J. R. DeWaard . 2003 Biological identifications through DNA barcodes. Proceedings of the Royal Society B 270:313–321. 12614582 10.1098/rspb.2002.2218 PMC1691236

[eap1914-bib-0043] Hebert, P. D. N. , E. H. Penton , J. M. Burns , D. H. Janzen , and W. Hallwachs . 2004a Ten species in one: DNA barcoding reveals cryptic species in the neotropical skipper butterfly *Astraptes fulgerator* . Proceedings of the National Academy of Sciences USA 101:14812–14817. 10.1073/pnas.0406166101 PMC522015 15465915

[eap1914-bib-0044] Hebert, P. D. N. , M. Y. Stoeckle , T. S. Zemlak , and C. M. Francis . 2004b Identification of birds through DNA barcodes. PLoS Biology 2:e312. 15455034 10.1371/journal.pbio.0020312 PMC518999

[eap1914-bib-0045] Hodgetts, J. , J. C. Ostoja‐Starzewski , T. Prior , R. Lawson , J. Hall , and N. Boonham . 2016 DNA barcoding for biosecurity: case studies from the UK plant protection program. Genome 59:1033–1048. 27792411 10.1139/gen-2016-0010

[eap1914-bib-0046] Hudson, M. E. 2008 Sequencing breakthroughs for genomic ecology and evolutionary biology. Molecular Ecology Resources 8:3–17. 21585713 10.1111/j.1471-8286.2007.02019.x

[eap1914-bib-0047] Iftikhar, R. , M. Ashfaq , A. Rasool , and P. D. N. Hebert . 2016 DNA barcode analysis of thrips (Thysanoptera) diversity in Pakistan reveals cryptic species complexes. PLoS ONE 11:e0146014. 26741134 10.1371/journal.pone.0146014 PMC4704811

[eap1914-bib-0048] Janzen, D. H. , M. Hajibabaei , J. M. Burns , W. Hallwachs , E. Remigio , and P. D. N. Hebert . 2005 Wedding biodiversity inventory of a large and complex Lepidoptera fauna with DNA barcoding. Philosophical Transactions of the Royal Society B 360:1835–1845. 10.1098/rstb.2005.1715 PMC1609230 16214742

[eap1914-bib-0049] Johnson, K. P. , B. L. Williams , D. M. Drown , R. J. Adams , and D. H. Clayton . 2002 The population genetics of host specificity: genetic differentiation in dove lice (Insecta: Phthiraptera). Molecular Ecology 11:25–38. 11903902 10.1046/j.0962-1083.2001.01412.x

[eap1914-bib-0050] Keane, R. M. C. , and M. J. Crawley . 2002 Exotic plant invasions and the enemy release hypothesis. Trends in Ecology & Evolution 17:164–170.

[eap1914-bib-0051] Kearse, M. , et al. 2012 Geneious basic: an integrated and extendable desktop software platform for the organization and analysis of sequence data. Bioinformatics 28:1647–1649. 22543367 10.1093/bioinformatics/bts199 PMC3371832

[eap1914-bib-0053] Kress, W. J. , K. J. Wurdack , E. A. Zimmer , L. A. Weigt , and D. H. Janzen . 2005 Use of DNA barcodes to identify flowering plants. Proceedings of the National Academy of Sciences USA 102:8369–8374. 10.1073/pnas.0503123102 PMC1142120 15928076

[eap1914-bib-0054] Kress, W. J. , D. L. Erickson , F. A. Jones , N. G. Swenson , R. Perez , O. Sanjur , and E. Bermingham . 2009 Plant DNA barcodes and a community phylogeny of a tropical forest dynamics plot in Panama. Proceedings of the National Academy of Sciences USA 106:18621–18626. 10.1073/pnas.0909820106 PMC2763884 19841276

[eap1914-bib-0055] Kress, W. J. , C. Garcia‐Robledo , M. Uriarte , and D. L. Erickson . 2015 DNA barcodes for ecology, evolution, and conservation. Trends in Ecology & Evolution 30:25–35. 25468359 10.1016/j.tree.2014.10.008

[eap1914-bib-0056] Leray, M. , C. P. Meyer , and S. C. Mills . 2015 Metabarcoding dietary analysis of coral dwelling predatory fish demonstrates the minor contribution of coral mutualists to their highly partitioned, generalist diet. PeerJ 3:e1047. 26137428 10.7717/peerj.1047 PMC4485734

[eap1914-bib-0057] Li, W. Z. , A. Cowley , M. Uludag , T. Gur , H. McWilliam , S. Squizzato , Y. M. Park , N. Buso , and R. Lopez . 2015 The EMBL‐EBI bioinformatics web and programmatic tools framework. Nucleic Acids Research 43:W580–W584. 25845596 10.1093/nar/gkv279 PMC4489272

[eap1914-bib-0058] Lobo, J. , M. A. L. Teixeira , L. M. S. Borges , M. S. G. Ferreira , C. Hollatz , P. T. Gomes , R. Sousa , A. Ravara , M. H. Costa , and F. O. Costa . 2016 Starting a DNA barcode reference library for shallow water polychaetes from the southern European Atlantic coast. Molecular Ecology Resources 16:298–313. 26129849 10.1111/1755-0998.12441

[eap1914-bib-0059] MacDougall, A. S. , B. Gilbert , and J. M. Levine . 2009 Plant invasions and the niche. Journal of Ecology 97:609–615.

[eap1914-bib-0060] Mazerolle, M. J. 2017 AICcmodavg: Model selection and multimodel inference based on (Q) AIC(c). R package version 2.1‐1. https://cran.r-project.org/package=AICcmodavg

[eap1914-bib-0061] Meyer, C. 2017 Moorea Biocode Project FASTA data. Merritt: Collection: Moorea Biocode Collection ark:/13030/m5478zfg. California Digital Library Version 1: 2016 ± 05 ± 14.

[eap1914-bib-0062] Meyer, C. P. , and G. Paulay . 2005 DNA barcoding: error rates based on comprehensive sampling. PLoS Biology 3:e422. 16336051 10.1371/journal.pbio.0030422 PMC1287506

[eap1914-bib-0063] Moritz, C. , and C. Cicero . 2004 DNA barcoding: Promise and pitfalls. PLoS Biology 2:e354. 15486587 10.1371/journal.pbio.0020354 PMC519004

[eap1914-bib-0064] Moulton, M. J. , H. Song , and M. F. Hiting . 2010 Assessing the effects of primer specificity on eliminating numt coamplification in DNA barcoding: a case study from Orthoptera (Arthropoda: Insecta). Molecular Ecology Resources 10:615–627. 21565066 10.1111/j.1755-0998.2009.02823.x

[eap1914-bib-0065] Nitta, J. H. 2008 Exploring the utility of three plastid loci for biocoding the filmy ferns (Hymenophyllaceae) of Moorea. Taxon 57:725–736.

[eap1914-bib-0066] Nitta, J. H. , J. Y. Meyer , and A. R. Smith . 2011 Pteridophytes of Mo'orea, French Polynesia: additional new records. American Fern Journal 101:36–49.

[eap1914-bib-0067] Oliveira, L. M. , T. Knebelsberg , M. Landi , P. Soares , M. J. Raupach , and F. O. Costa . 2015 Compilation and validation of a global DNA barcode reference library for European marine fishes. Genome 58:262–263. 10.1111/jfb.13169 27739061

[eap1914-bib-0068] Oliveira, L. M. , T. Knebelsberger , M. Landi , P. Soares , M. J. Raupach , and F. O. Costa . 2016 Assembling and auditing a comprehensive DNA barcode reference library for European marine fishes. Journal of Fish Biology 89:2741–2754. 27739061 10.1111/jfb.13169

[eap1914-bib-0069] Park, D. S. , J. H. Jeon , B. K. Byun , K. J. Hong , S. H. Lee , and D. P. Ryu . 2015 A DNA barcode reference library for Asian quarantine pests. Genome 58:264–265.

[eap1914-bib-0052] Parker, M. , S. Stones‐Havas , C. Starger , and C. Meyer . 2012 Laboratory information management systems for DNA barcoding. Methods in Molecular Biology 858:269–310. 22684961 10.1007/978-1-61779-591-6_13

[eap1914-bib-0070] Pejovic, I. , A. Ardura , L. Miralles , A. Arias , Y. J. Borrell , and E. Garcia‐Vazquez . 2016 DNA barcoding for assessment of exotic molluscs associated with maritime ports in northern Iberia. Marine Biology Research 12:168–176.

[eap1914-bib-0071] Pennisi, E. 2017 Sequencing all life captivates biologists. Science 355:894. 28254891 10.1126/science.355.6328.894

[eap1914-bib-0072] Pilgrim, E. M. , S. A. Jackson , S. Swenson , I. Turcsanyi , E. Friedman , L. Weigt , and M. J. Bagley . 2011 Incorporation of DNA barcoding into a large‐scale biomonitoring program: opportunities and pitfalls. Journal of the North American Benthological Society 30:217–231.

[eap1914-bib-0073] Pisa, S. , A. Vanderpoorten , J. Patiño , O. Werner , J. M. González‐Mancebo , and R. M. Ros . 2015 How to define nativeness in vagile organisms: lessons from the cosmopolitan moss *Bryum argenteum* on the island of Tenerife (Canary Islands). Plant Biology 17:1057–1065. 25980839 10.1111/plb.12348

[eap1914-bib-0074] Pomerantz, A. , N. Penafiel , A. Arteaga , L. Bustamante , F. Pichardo , L. A. Coloma , C. L. Barrio‐Amoros , D. Salazar‐Valenzuela , and S. Prost . 2018 Real‐time DNA barcoding in a rainforest using nanopore sequencing: opportunities for rapid biodiversity assessments and local capacity building. Gigascience 7:giy033. 10.1093/gigascience/giy033 PMC5905381 29617771

[eap1914-bib-0075] Pons, J. , T. G. Barraclough , J. Gomez‐Zurita , A. Cardoso , D. P. Duran , S. Hazell , S. Kamoun , W. D. Sumlin , and A. P. Vogler . 2006 Sequence‐based species delimitation for the DNA taxonomy of undescribed insects. Systematic Biology 55:595–609. 16967577 10.1080/10635150600852011

[eap1914-bib-0076] Porco, D. , T. Decaens , L. Deharveng , S. W. James , D. Skarzynski , C. Erseus , K. R. Butt , B. Richard , and P. D. N. Hebert . 2013 Biological invasions in soil: DNA barcoding as a monitoring tool in a multiple taxa survey targeting European earthworms and springtails in North America. Biological Invasions 15:899–910.

[eap1914-bib-0077] Puillandre, N. , A. Lambert , S. Brouillet , and G. Achaz . 2012 ABGD, automatic barcode gap discovery for primary species delimitation. Molecular Ecology 21:1864–1877. 21883587 10.1111/j.1365-294X.2011.05239.x

[eap1914-bib-0078] R Core Team . 2014 R: A language and environment for statistical computing. R Foundation for Statistical Computing, Vienna, Austria http://www.R-project.org/

[eap1914-bib-0079] Ramage, T. 2017 Checklist of the terrestrial and freshwater arthropods of French Polynesia (Chelicerata; Myriapoda; Crustacea; Hexapoda). Zoosystema 39:213–225.

[eap1914-bib-0080] Ramage, T. , P. Martins‐Simoes , G. Mialdea , R. Allemand , A. Duplouy , P. Rousse , N. Davies , G. K. Roderick , and S. Charlat . 2017 A DNA barcode‐based survey of terrestrial arthropods in the Society Islands of French Polynesia: host diversity within the SymbioCode Project. European Journal of Taxonomy 272:1–13.

[eap1914-bib-0081] Ratnasingham, S. , and P. D. N. Hebert . 2007 BOLD: the barcode of life data system ( http://www.barcodinglife.org). Molecular Ecology Notes 7:355–364. 18784790 10.1111/j.1471-8286.2007.01678.x PMC1890991

[eap1914-bib-0082] Rimet, F. , P. Chaumeil , F. Keck , L. Kermarrec , V. Vasselon , M. Kahlert , A. Franc , and A. Bouchez . 2016 R‐Syst:diatom: an open‐access and curated barcode database for diatoms and freshwater monitoring. Database: The Journal of Biological Databases and Curation 2016:baw016. 26989149 10.1093/database/baw016 PMC4795936

[eap1914-bib-0083] Rubinoff, D. , S. Cameron , and K. Will . 2006 A genomic perspective on the shortcomings of mitochondrial DNA for “barcoding” identification. Journal of Heredity 97:581–594. 17135463 10.1093/jhered/esl036

[eap1914-bib-0084] Saunders, G. W. 2009 Routine DNA barcoding of Canadian Gracilariales (Rhodophyta) reveals the invasive species *Gracilaria vermiculophylla* in British Columbia. Molecular Ecology Resources 9:140–150. 10.1111/j.1755-0998.2009.02639.x 21564973

[eap1914-bib-0085] Savage, J. , P. D. N. Hebert , and V. Levesque‐Beaudin . 2015 The Muscidae of Canada: towards a complete DNA barcode reference library. Genome 58:277.

[eap1914-bib-0086] Savolainen, V. , R. S. Cowan , A. P. Vogler , G. K. Roderick , and R. Lane . 2005 Towards writing the encyclopaedia of life: an introduction to DNA barcoding. Philosophical Transactions of the Royal Society B 360:1805–1811. 10.1098/rstb.2005.1730 PMC1609222 16214739

[eap1914-bib-0087] Smith, M. A. , and B. L. Fisher . 2009 Invasions, DNA barcodes, and rapid biodiversity assessment using ants of Mauritius. Frontiers in Zoology 6:31. 20003263 10.1186/1742-9994-6-31 PMC2804717

[eap1914-bib-0088] Stein, E. D. , B. P. White , R. D. Mazor , J. K. Jackson , J. M. Battle , P. E. Miller , E. M. Pilgrim , and B. W. Sweeney . 2014 Does DNA barcoding improve performance of traditional stream bioassessment metrics? Freshwater Science 33:302–311.

[eap1914-bib-0089] Strauss, S. Y. , C. O. Webb , and N. Salamin . 2006 Exotic taxa less related to native species are more invasive. Proceedings of the National Academy of Sciences USA 103:5841–5845. 10.1073/pnas.0508073103 PMC1421337 16581902

[eap1914-bib-0090] Teasdale, S. E. , A. K. Beulke , P. L. Guy , and D. A. Orlovich . 2013 Environmental barcoding of the ectomycorrhizal fungal genus *Cortinarius* . Fungal Diversity 58:299–310.

[eap1914-bib-0091] Thalmann, O. , J. Hebler , H. N. Poinar , S. Paabo , and L. Vigilant . 2004 Unreliable mtDNA data due to nuclear insertions: a cautionary tale from analysis of humans and other great apes. Molecular Ecology 13:321–335. 14717890 10.1046/j.1365-294x.2003.02070.x

[eap1914-bib-0092] Thomas, V. G. , R. H. Hanner , and A. V. Borisenko . 2016 DNA‐based identification of invasive alien species in relation to Canadian federal policy and law, and the basis of rapid‐response management. Genome 59:1023–1031. 27767334 10.1139/gen-2016-0022

[eap1914-bib-0093] Tixier, M.‐S. , F. A. Hernandes , S. Guichou , and S. Kreiter . 2011 The puzzle of DNA sequences of Phytoseiidae (Acari: Mesostigmata) in the public GenBank database. Invertebrate Systematics 25:389–406.

[eap1914-bib-0094] Valentini, A. , F. Pompanon , and P. Taberlet . 2009 DNA barcoding for ecologists. Trends in Ecology & Evolution 24:110–117. 19100655 10.1016/j.tree.2008.09.011

[eap1914-bib-0095] Wägele, H. 2011 The taxonomist – an endangered race. A practical proposal for its survival. Frontiers in Zoology 18:25. 10.1186/1742-9994-8-25 PMC3210083 22029904

[eap1914-bib-0096] Wallace, A. R. 1880 Island life: or, the phenomena and causes of insular faunas and floras, including a revision and attempted solution of the problem of geological climates. Macmillan, London, UK.

[eap1914-bib-0097] Warne, C. P. K. , S. L. deWaard , J. R. Kohn , J. P. Rebman , M. L. Kuzmina , and B. A. Zlotnick . 2015 DNA barcoding the plants of San Diego County, California: on the verge of the first complete DNA barcode reference library for a globally important regional flora. Genome 58:295.

[eap1914-bib-0098] Wheeler, Q. 2014 Are reports of the death of taxonomy an exaggeration? New Phytologist 201:370–371. 26013181 10.1111/nph.12612

[eap1914-bib-0099] Xu, C. , W. P. Dong , S. Shi , T. Cheng , C. H. Li , Y. L. Liu , P. Wu , H. K. Wu , P. Gao , and S. L. Zhou . 2015 Accelerating plant DNA barcode reference library construction using herbarium specimens: improved experimental techniques. Molecular Ecology Resources 15:1366–1374. 25865498 10.1111/1755-0998.12413

[eap1914-bib-0100] Yang, Y. Z. , A. B. Zhan , L. Cao , F. J. Meng , and W. B. Xu . 2016 Selection of a marker gene to construct a reference library for wetland plants, and the application of metabarcoding to analyze the diet of wintering herbivorous waterbirds. PeerJ 4:e2345. 27602302 10.7717/peerj.2345 PMC4991844

