# Supplementary material for: Categorization of species as native or nonnative using DNA sequence signatures without a complete reference library
Source: Ecol Appl. 2019 Jun 12;29(5):e01914. doi: 10.1002/eap.1914 (PMC7079013; doi:10.1002/eap.1914)
Supplement: Supplementary file 1 [file EAP-29-e01914-s001.zip › eap1914-sup-0001-AppendixS1.pdf]

Supporting Information. Andersen, J.C., P. Oboyski, N. Davies, S. Charlat, C. Ewing, C. Meyer, H. Krehenwinkel, J. Y. Lim, S. Noriyuki, T. Ramage, R. G. Gillespie, and G. K. Roderick. 2019. Categorization of species as likely native or likely non-native using DNA sequence signatures without a complete reference library. *Ecological Applications.*

Appendix S1

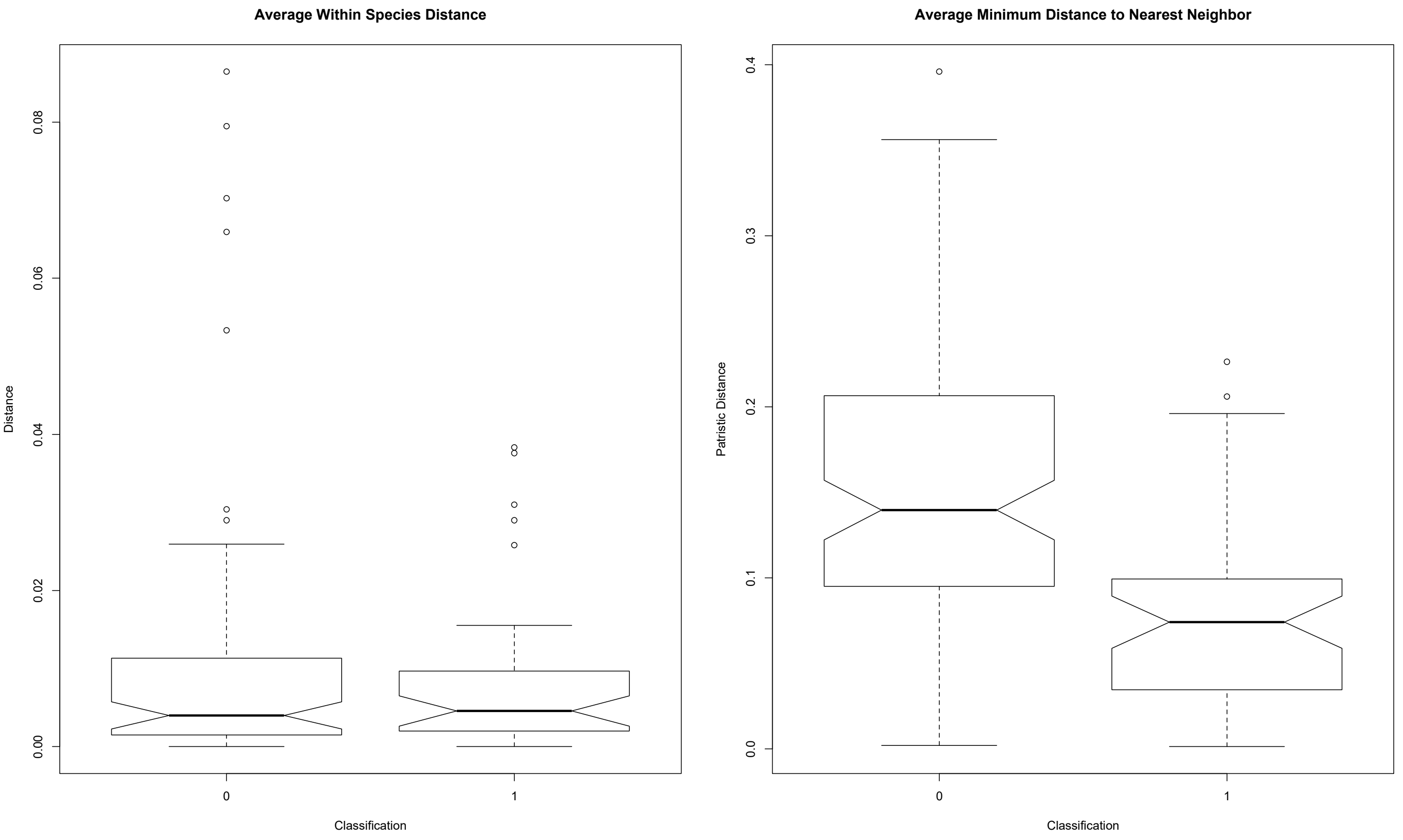

Fig. S1. Metrics of within species and between species differences based on known endemic and known introduced species in the training dataset. Boxplots are drawn using the default settings in R, with dark horizontal lines represent the median values, whiskers representing 1.5 x the interquartile range, outliers drawn as open circles, and notches drawn to represent approximations of 95% CIs.
