# Supplementary material for: Categorization of species as native or nonnative using DNA sequence signatures without a complete reference library
Source: Ecol Appl. 2019 Jun 12;29(5):e01914. doi: 10.1002/eap.1914 (PMC7079013; doi:10.1002/eap.1914)
Supplement: Supplementary file 1 [file EAP-29-e01914-s001.zip › eap1914-sup-0003-MetadataS1.pdf]

Andersen, J.C., P. Oboyski, N. Davies, S. Charlat, C. Ewing, C. Meyer, H. Krehenwinkel, J. Y. Lim, S. Noriyuki, T. Ramage, R. G. Gillespie, and G. K. Roderick. 2019. Categorization of species as likely native or likely non-native using DNA sequence signatures without a complete reference library. *Ecological Applications.*

Data S1

Supporting data and custom R script.

Authors

Jeremy C. Andersen
Department of Environmental Science Policy and Management
130 Mulford Hall, University of California Berkeley, Berkeley, CA 94720-3114
jandersen@berkeley.edu

Peter Oboyski
Essig Museum of Entomology
University of California Berkeley, Berkeley, CA 94720
poboyski@berkeley.edu

Neil Davies
Gump South Pacific Research Station
University of California Berkeley, Maharepa, Moorea, French Polynesia
ndavies@berkeley.edu

Sylvain Charlat
Biométrie et Biologie Évolutive
UMR CNRS, 69622 Villeurbanne, France
sylvain.charlat@univ-lyon1.fr

Curtis Ewing
Komohana Research and Extension Center
University of Hawai'i at Mānoa, Hilo, HI 96720
cpe1@hawaii.edu

Christopher Meyer
Smithsonian Institution
Washington, DC 20013
meyerc@si.edu

Henrik Krehenwinkel
Department of Biogeography
Universität Trier, Germany
krehenwinkel@uni-trier.de

Jun Ying Lim
Department of Integrated Biology
3040 Valley Life Sciences Bldg #3140, Berkeley CA 94720
junyinglim@berkeley.edu

Suzuki Noriyuki
Faculty of Agriculture and Marine Science
Kochi University, Japan
fvgnoriyuki@gmail.com

Thibault Ramage
Quartier de la Glacière
29900 Concarneau, France
thibault.ramage@hotmail.fr

Rosemary G. Gillespie
Department of Environmental Science Policy and Management
130 Mulford Hall, University of California Berkeley, Berkeley, CA 94720-3114
gillespie@berkeley.edu

George K. Roderick
Department of Environmental Science Policy and Management
130 Mulford Hall, University of California Berkeley, Berkeley, CA 94720-3114
roderick@berkeley.edu

File list (files found within DataS1.zip)

```
CollectionInformation.csv
RamageChecklist.csv
CustomRscript.R
SummaryGeneticInformation.csv
```

Description

`CollectionInformation.csv` — Collection, sequence, and best-match information for all specimens that were retained after both length and contamination filters.

`RamageChecklist.csv` — Ramage (2017) checklist for the terrestrial insects and spiders of French Polynesia.

`CustomRscript.R` — Custom R script for calculating within species average similarity and minimum distances between species based, and for conducting GLMM estimation of the native/non-native status of species.

`SummaryGeneticInformation.csv` — Summary information for each species, categorization according to Ramage (2017), nucleotide diversity statistics for each species (including average percent similarity, average within species distances, root branch length, and average patristic distance to nearest neighbor species), GLMM results, and predicted categorization based on GLMM (either "Native", "Non-Native", "Undet", or "NA").
